# Fermentation products of Danshen relieved dextran sulfate sodium-induced experimental ulcerative colitis in mice

**DOI:** 10.1038/s41598-021-94594-7

**Published:** 2021-08-10

**Authors:** Le Su, Yue Su, Zaiyong An, Ping Zhang, Qiulin Yue, Chen Zhao, Xin Sun, Song Zhang, Xinli Liu, Kunlun Li, Lin Zhao

**Affiliations:** 1 State Key Laboratory of Biobased Material and Green Papermaking, School of Bioengineering, Qilu University of Technology, Shandong Academy of Sciences, Jinan, 250353 China; 2 Shandong Provincial Key Laboratory of Food and Fermentation Engineering, Shandong Food Ferment Industry Research and Design Institute, Qilu University of Technology, Shandong Academy of Sciences, Jinan, 250013 China; 3 Jinan Hangchen Biotechnology Co., Ltd., Jinan, 250353 China

**Keywords:** Chemical biology, Microbiology

## Abstract

With the increased incidence and recognition, ulcerative colitis (UC) has become a global public health problem in the world. Although many immunosuppressant and biological drugs have been used for UC treatment, the cure rate is still very low. It is necessary to find some safe and long-term used medicine for UC cure. Recently, the Chinese traditional herb Danshen has been investigated in the treatment of UC. However, it is a limitation of Danshen that many of the active components in Danshen are not easily absorbed by the human body. Probiotics could convert macromolecules into smaller molecules to facilitate absorption. Thus, *Lactobacillus rhamnosus* (F-B4-1) and *Bacillus subtillis Natto* (F-A7-1) were screened to ferment Danshen in this study. The fermented Danshen products were gavaged in the dextran sulfate sodium (DSS)-induced UC model mice. Danshen had better results to attenuate symptoms of DSS-induced UC after fermented with F-B4-1 and F-A7-1. Loss of body weight and disease activity index (DAI) were reduced. The abnormally short colon lengths and colonic damage were recovered. And fermented Danshen had the better inhibitory effect than Danshen itself on pro-inflammatory cytokine expression during DSS-induced UC. The results indicated that compared with Danshen, fermented Danshen relieved DSS-induced UC in mice more effectively. Danshen fermented by probiotics might be an effective treatment to UC in clinic stage in the future.

## Introduction

Ulcerative colitis (UC) is a non-specific intestinal inflammatory disease characterized by a chronic inflammatory reaction and intestinal mucosal epithelial damage. The major clinical characteristics of the UC is abdominal pain, blood in the stool, diarrhea, and weight loss. Thus, the quality of life of patients with UC declines^1^. Although it has been identified that several factors contribute to UC, the currently available therapies are limited^2^. Now in clinical treatment, aminosalicylates, topical and systemic steroids, immunosuppressants and biological drugs are used to treat UC^3^. However, low cure rate and high recurrence rate are two major problems of UC treatment drugs^4^. And the incidence and prevalence of UC have been increasing over time. Intermittent or transitory therapy would lead to repeated symptoms and eventually lead to structure or function damage of the intestinal. While long-term or high-dose use of the UC drugs would result in many side effects, such as abdominal pain, kidney damage, hepatotoxicity and blood disorders^5^. Thus, it is necessary to find some new drugs to cure UC.

*Salvia miltiorrhiza Bunge* (Danshen) is a traditional herb that has been used in China for more than 2,000 years. Danshen has a wide spectrum of pharmacological activities, such as anti-inflammatory, anticancer, cardiovascular treatment, anti-diabetes and so on^6–9^. It has been reported that a variety of natural ingredients in Danshen could protect dextran sulfate sodium (DSS)-induced experimental UC in mice, such as Salvianolic acid B , tanshinone IIA, Danshensu and dihydrotanshinone I^10–12^. The effective components of Danshen, fat-soluble tanshinone and water-soluble phenolic acid could regulate microbial disorder and have a protective effect on experimental colitis induced by DSS in mice^13,14^. Salvianolic acid B the active component extracted from Danshen could increase the content of small intestinal short-chain fatty acids, increase the diversity of intestinal flora, improve intestinal microbial dysfunction, and prevent and protect the occurrence of inflammatory bowel disease^15^. Salvianolic acid A could significantly down-regulate the expression of IL-1β, MCP-1 and IL-6 genes in colon tissue of DSS-induced colitis model mice, showing potential therapeutic value for colitis^16^. Although the extensive pharmacological effects of Danshen have been fully confirmed by researchers, many of the active components in Danshen are not easily absorbed by the human body, such as Danshensu. Due to the structural characteristics of Danshensu with o-phenol hydroxyl and α-hydroxyl carboxylic acid, it has poor lipid solubility, unstable properties, easy oxidation and low oral bioutilization which reduced the utilization rate. This is a limitation of a direct application of Danshen.

Gut modulation by probiotics would be one potential strategy to prevent or treat UC. Over the past twenty years, many investigations have focused on the role of probiotics in the treatment of UC^17^, including *Bifidobacterium animalis* subsp. *lactis* BB12^18^, *Bifidobacterium longum* subsp. *infantis* BB‐02^19^, *Lactobacillus rhamnosus* CNCM I-3690^20^, *Lactobacillus*^21^, *Lactobacillus rhanosum*^22,23^ and so on. Not only one probiotic, even the probiotic mixture had the better effects. Until now, the most known probiotic mixture with proven efficacy is VSL#3. This mixture contains 900 billion lyophilized bacteria, including four strains of *Lactobacillus*, three strains of *Bifidobacteria* and one strain of *Streptococcus thermophilus*^24,25^. In addition to improving the gut flora, probiotics could also convert macromolecules into smaller molecules to facilitate absorption. They could not only decompose polysaccharides that are difficult to digest into monosaccharides and lactic acid, which could promote the absorption of active substances in the intestines, but also degrade macromolecular proteins into polypeptides and amino acids, which reduced the burden of protein degradation in the intestines and stomach. In addition, the use of probiotics for fermentation could also convert unstable substances into stable derivatives for better production and utilization^26^. Thus, it is a good alternative method to make the active molecules in Danshen to have better pharmacological activity, strong stability, fat solubility and high bioavailability by biological fermentation^27^. However whether the efficacy of Danshen could be enhanced by fermentation has not been reported.

Both *Lactobacillus rhamnosus* and *Bacillus natto* are probiotics, which could maintain the ecology of human intestinal flora after oral administration. They also played a key role in the proliferation and differentiation of intestinal epithelial cells as well as the development and homeostasis of the immune system^28^. In both sterile and normal mice, *Lactobacillus rhamnosus* could promote the growth and differentiation of small intestinal cells, increase the crypt depth of intestinal epithelium, lengthen the length of small intestinal villi and increase the number of villi cells^29^. *Bacillus subtilis* could protect the integrity of intestinal epithelial cells, up-regulate the expression of ZO-1 and Occludin, reduce the damage of intestinal epithelial cells, and enhance the barrier function of intestinal epithelial cells^30,31^.

In this manuscript, the screened *Lactobacillus rhamnosus* (F-B4-1) and *Bacillus subtillis Natto* (F-A7-1) were used to ferment Danshen. The effects of the fermentation products on UC were investigated. It is proved that after fermented by probiotics, Danshen had the better results to attenuate symptom of DSS-induced UC by anti-inflammatory effect. Therefore, the Danshen fermented by probiotics might be an effective drug to treat UC in the clinic in the future.

## Methods

### Chemicals and reagents

Human and mouse IL-6, TNF-α and IL-1β ELISA kit were purchased from Beijing dakowei Biotechnology Co., Ltd. Lipopolysaccharide (LPS) (*Escherichia coli* 055:B5) from Sigma–Aldrich (St. Louis, MO) was dissolved in ddH_2_O as a stock solution at a concentration of 1 mg/mL. Danshen was purchased from Shandong Institute of Traditional Chinese Medicine. Acid cellulase, acid pectinase, whey protein powder, peptone, Mann Rogosa Sharp (MRS, lactic acid bacteria) liquid medium, MRS solid medium, nutrient liquid medium (Dried grass), LB liquid medium (*Staphylococcus aureus *(*S. aureus*),* Escherichia coli *(*E. coli*)), LB solid medium (*S. aureus*,* E. coli*), liquid medium for Salmonella (*Salmonella typhimurium *(*S. Typhimurium*)* and Salmonella enteritidis *(*S. Enteritidis*)) and salmonella solid culture medium (*S. Typhimurium and S. Enteritidis*) were from Solebol Technology Co. LTD.. DSS (MW 40000) was obtained from Shanghai Aladdin Biochemical Technology Co., LTD (Shanghai China).

### Cell culture and treatment

Caco-2 cells (presented by professor Yanqing Li from Qilu hospital of China) were cultured in Dulbecco’s Modified Eagle’s Medium-Hight glucose (DMEM-H, Gibco, 12800-017) supplemented with 10% fetal bovine serum (FBS; v/v) (Hyclone, SV30087.02). Cells were kept at 37 °C in a humidified atmosphere of 5% CO_2_ under standard conditions. When caco-2 cells were grown to 80% confluency, cells were stimulated with LPS. Sample treatments were done 12 h after cells exposure to LPS.

### Cell viability analysis

Cell viability was determined by MTT (3-[4,5-dimethylthiazol-2-yl]-2,5-diphenyltertrazolium bromide) assay^32^. Cells were seeded into 96-well plates and treated with LPS. After 12 h LPS treatment, cells were treated with or without different samples. Then cells were incubated with 0.5% MTT for 4 h. The medium was removed and 100 μL of 0.04 M dimethylsulfoxide solution (DMSO) was added. The absorbance of the reaction product in solution at 570 nm was measured using a SpectraMax ABS microplate spectrophotometer (Molecular Devices, USA). The percentage of living cells was calculated by the ratio of OD.

### Antimicrobial spectrum analysis

Four pathogens (*S. aureus*,* E. coli*,* S. Typhimurium and S. Enteritidis*) were selected and inoculated with LB medium (L3522, Sigma-Aldrich) and Salmonella medium (peptone 5 g, beef extract 3 g, NaCl 50 g, pH 7.0–7.2) respectively at 37 °C for 12 h. Then absorb the pathogen bacteria suspension (200 μL) into agar culture-medium (20 mL) and mix. The mixture was placed at room temperature for 1 h. Oxford cup method was used to determine the bacteriostatic ability of the strain. And 4 sterile Oxford cups were placed in the petri dish at a medium distance. Nine kinds of probiotics (*Lactobacillus rhamnosus* (F-B4-1), *Lactobacillus plantarum* (F-B8-1), *Lactobacillus fermentans* (F-B9-1), *Streptococcus thermophilus* (F-B12-1), *Lactobacillus casei* (F-B16-1),* Leuconostoc enterococcus* (F-B20-1),* Lactobacillus Harbin* (F-B25-1),* Bacillus subtilis* (F-A1-2),* Bacillus subtillis Natto* (F-A7-1)) were selected and inoculated with MRS medium (peptone 10 g, beef extract 10 g, yeast powder 5 g, glucose 20 g, ammonium citrate 2 g, MgSO_4_·7H_2_O 0.58 g, CH_3_COONa 3.12 g, Na_2_HPO_4_ 1.63 g, CH_3_COOK 2.75 g, MnSO_4_·4H_2_O 0.25 g, twain 80 1 mL) and nutrient medium (yeast extract 10 g, beef extract 10 g, peptone 5 g, glucose 10 g, NaCl 5 g, pH 7.9) respectively at 37 °C for 12 h. The viable counts of all probiotics were above 1 × 10^8^ CFU/mL. The supernatants (50μL) of the nine different strains were added into each Oxford cup, and then diffused at 4 °C for 6 h and cultured at 37 °C for 48 h. The antibacterial circles around the Oxford cup were observed and their diameters were measured^33^. Its diameter was analyzed and measured using ImageJ software. The liquid medium was as the blank control.

### Gastric and intestinal fluid tolerance

The bacterial strain was inoculated in 5 mL MRS liquid medium for 24 h at 37 °C. After centrifuged at 3000 rpm for 10 min, the supernatant was discarded and rinsed with PBS buffer (pH 7.0) for 3 times. After suspended the bacterial in 5 mL sterile saline, 1 mL bacterial suspension was mixed with 9 mL artificial gastric juice (NaCl 0.2 g, pepsin 0.35 g, ddH_2_O 100 mL, pH 2.5) or artificial intestinal juice (trypsin 0.1 g, NaHCO_3_ 1.1 g, NaCl 0.2 g, bile salt 1.8 g, ddH_2_O 100 mL, pH 8.0) and cultured at 37 °C for 3 h or 4 h. Then, the culture medium was diluted with normal saline. 1 mL diluent was mixed with 30 mL MRS solid medium and cultured at 37 °C for 48 h. To determine the bacterial survival, viable cell counts were determined at 0 h, 3 h or 4 h of incubation. The survival rate was calculated by counting. Percentage survival was calculated using the formula^34^:

$$\mathrm{\%survival}=\frac{\mathrm{log}CFU\; N1}{\mathrm{log}CFU\; N0}\times 100$$

where N1 is the viable count after exposure to simulated gastrointestinal fluids for specific time intervals and N0 represents cell count before treatment^35^.

### The treatments of Danshen

All plant experiments complied with the IUCN Policy Statement on Research Involving Species at Risk of Extinction and the Convention on the Trade in Endangered Species of Wild Fauna and Flora. The Danshen was divided into three different treatments, boiled, enzymolysis and fermentation. (1) *Boiled* Ultramicro mill was used to crush Danshen into powder above 1500 mesh. The ratio of the powder and distilled water was 1:9. After the uniform stirring, the water was extracted by boiling at 100 °C for 30 min. And then the water was filled up. The sample was centrifuged at 10,000 r/min and the supernatant was reserved. (2) *Enzymolysis* The Danshen was ultra-fine pulverized and dissolved in sterile water (1:9, pH 4.8–5.0) after ultraviolet irradiation. The enzymolysis was did at 50 °C for 2 h by adding acid pectinase and acid cellulase (acid fiber and sour fruit amount accounted for 1 ‰ of solid). Then sugar (2%), peptone (5 ‰ of total volume) and protein powder (2.5 ‰ of total volume) were added. The samples were pasteurization (85 °C for 30 min) to form raw material culture medium. (3) *Fermentation* After enzymolysis, Denshen was fermented by four different manners. FDS47 (Fermented Danshen Sequence 47): Fermented Danshen with F-B4-1 (shaking table) at 37 °C for 12 h until the concentration of the reducing sugar was stable (pH 6.5–7.0). The number of colonies of F-B4-1was counted (1.2 × 10^9^ CFU/mL). Then it was further fermented with F-A7-1 (static culture) at 37 °C for 12 h until the concentration of the reducing sugar was stable. The numbers of colonies of F-B4-1and F-A7-1were counted (2 × 10^8^ CFU/mL). FDS74 (Fermented Danshen Sequence 74): Fermented Danshen with F-A7-1 (shaking table) at 37 °C for 12 h until the concentration of the reducing sugar was stable (pH 6.5–7.0). The number of colonies of F-A7-1was counted (8 × 10^8^ CFU/mL). Then it was further fermented with F-B4-1 (static culture), at 37 °C for 12 h until the concentration of the reducing sugar was stable. The numbers of colonies of F-B4-1 and F-A7-1 were counted (6 × 10^8^ CFU/mL). FDT47-sha (Fermented Danshen Together 47 Shake): Fermented Danshen with F-B4-1 and F-A7-1 together (shaking table) at 37 °C for 12 h until the concentration of the reducing sugar was stable (pH 6.5–7.0). The numbers of colonies of F-B4-1 and F-A7-1 were counted (1.53 × 10^9^ CFU/mL). FDT47-sta (Fermented Danshen Together 47 Stationary): Fermented Danshen with F-B4-1 and F-A7-1 together (static culture) at 37 °C for 12 h until the concentration of the reducing sugar was stable (pH 6.5–7.0). The numbers of colonies of F-B4-1 and F-A7-1 were counted (3.3 × 10^8^ CFU/mL).

All the samples were filtered through a 60 μm nylon net filter (Millipore, Bedford, MA, USA) and freeze-dried. The yields of solid content were FDS47-sha 5.8%, FDS47 5.7%, FDS74 5.7% and FDS47-sta 5.1%. The freeze-dried powder was dissolved in ddH_2_O. The solution was filtered (0.2 μm, pore size) and maintained at 4 °C prior to use.

### Microbial counts

The fermented Danshen liquid was vortexed to homogenize and the supernatant was obtained. For counting F-B4-1, 0.01 mL of the diluted samples was spread onto a MRS solid medium. Plates were prepared in duplicate and incubated at 37 °C for 24 h. For counting F-A7-1, 0.1 mL of the diluted samples was spread onto a nutrient solid medium. The plates were incubated at 37 °C for 24 h, and the viable cells were enumerated. Cell counts were expressed as CFU/mL.

### Animal treatment

Female C57BL/6 mice (6-weeks-old) were purchased from Beijing Vital River Laboratory Animal Technology Co., Ltd.. Mice were housed under standard conditions of humidity, room temperature and dark–light cycles. All animal experiments complied with the ARRIVE guidelines and were carried out in accordance with the U.K. Animals (Scientific Procedures) Act, 1986 and associated guidelines, EU Directive 2010/63/EU for animal experiments and the National Institutes of Health guide for the care and use of Laboratory animals (NIH Publications No. 8023, revised 1978). The animal experimental protocol complied with the Animal Management Rules of the Chinese Ministry of Health (document no. 55, 2001) and was approved by the Animal Experiment Ethnics Committee of Qilu University of Technology. All animals were divided into 5 groups (n = 7 per group). Mice were treated with saline for 15 days in Negtive control groups (NC), DSS, Boiled groups (Boi), compound bacterium fluid groups (CBF, the mixture of F-B4-1 and F-A7-1 in 1:1 ratio) and FDS47 groups were treated with drinking water containing 2.5% DSS for 7 days^36^. On the 8th day, mice were treated daily with saline, Boiled Danshen (50 mg/Kg), CBF or FDS47 (50 mg/Kg) by gavage respectively in the DSS, Boi, CBF and FDS47 groups. The probiotics of F-B4-1 and F-A7-1 were inoculated as described in the “Antimicrobial spectrum analysis”. Every mice was gavaged with 200 μL samples in the experiment. All mice were weighed every day. On the 15th day of the experiment, all mice were killed by exsanguination after deep anesthesia^37,38^.

### Evaluation of colitis

The disease activity index (DAI) was determined by an investigator blinded to the protocol by scoring changes in weight, hemoccult positivity or gross bleeding, and stool consistency as the protocol previously described^36^.

### Blood and tissue collection

Serum was prepared by centrifugation at 3000*g* for 20 min at 4 °C and stored at − 80 °C for biochemical analysis. The mice were killed by exsanguination after deep anesthesia. Colons were rapidly removed and their length were documented. Then the colons were snap-frozen in optimal cutting temperature (OCT) embedding medium (Tissue-Tek) for histology analysis.

### Hematoxylin and eosin (H&E) analyses

The distal colon were dissected and immersed in OCT embedding medium. Serial 8-μm-thick cryosections from every 3 sections (8–10 sections per mouse) were mounted on poly-d-lysine-coated slides. Cryosections were prepared and stained with H&E. Each colon was calculated from 4 consecutive 8-μm sections taken every 40 μm and covering the colon. H&E staining was done in a blinded manner. And the degree of inflammation and epithelial damage on microscopic H&E staining sections (8 µm) of distal colon was graded according to the method of Hudert and coworkers^39^. Briefly, the inflammation score is a combined score of (i) severity of inflammation: 0 (no inflammation); 1 (mild); 2 (moderate); 3 (severe) and (ii) thickness of inflammatory cell infiltration: 0 (no inflammation); 1 (mucosa); 2 (mucosa plus submucosa); 3 (transmural) and epithelial damage score consisting of character: 0 (intact epithelium); 1 (disruption of architectural structure); 2 (erosion); 3 (ulceration) and extent: 0 (no lesions); 1 (punctuate); 2 (multifocal); 3 (diffuse) of lesions. The total highest score in this study was 10.

### Enzyme-linked immunosorbent assays (ELISA)

The mouse intestinal tissues were homogenized on ice with NP40 lysis buffer (Beyotime Biotechnology, China). The homogenates were quantified using the BCA assay as reported previously (Beyotime Biotechnology, China)^40^. Cell supernatants, serum and tissue homogenates were collected respectively for the determination of IL-6, IL-1β and TNF-α concentrations according to the manufacturers’ instructions.

### Statistical analysis

All experiments were repeated at least 3 times independently. The normal distribution was firstly analyzed by SPSS v11.5 (SPSS Inc., Chicago, IL). All the data were expressed as mean ± SEM and analyzed with one-way ANOVA with use of SPSS v11.5 to compare the treatment means when data was normally distributed. Tukey–Kramer multiple comparison procedure was used for Post-Hoc comparisons. *P* values < 0.05 were considered statistically significant. Images were processed by use of Graphpad Prism 5 (GraphPad Software, La Jolla, CA, USA) and Adobe Photoshop CS6 (Adobe, San Jose, USA).

## Results

### F-B4-1 and F-A7-1 were selected to ferment enzymatic Danshen

**Figure 1:**
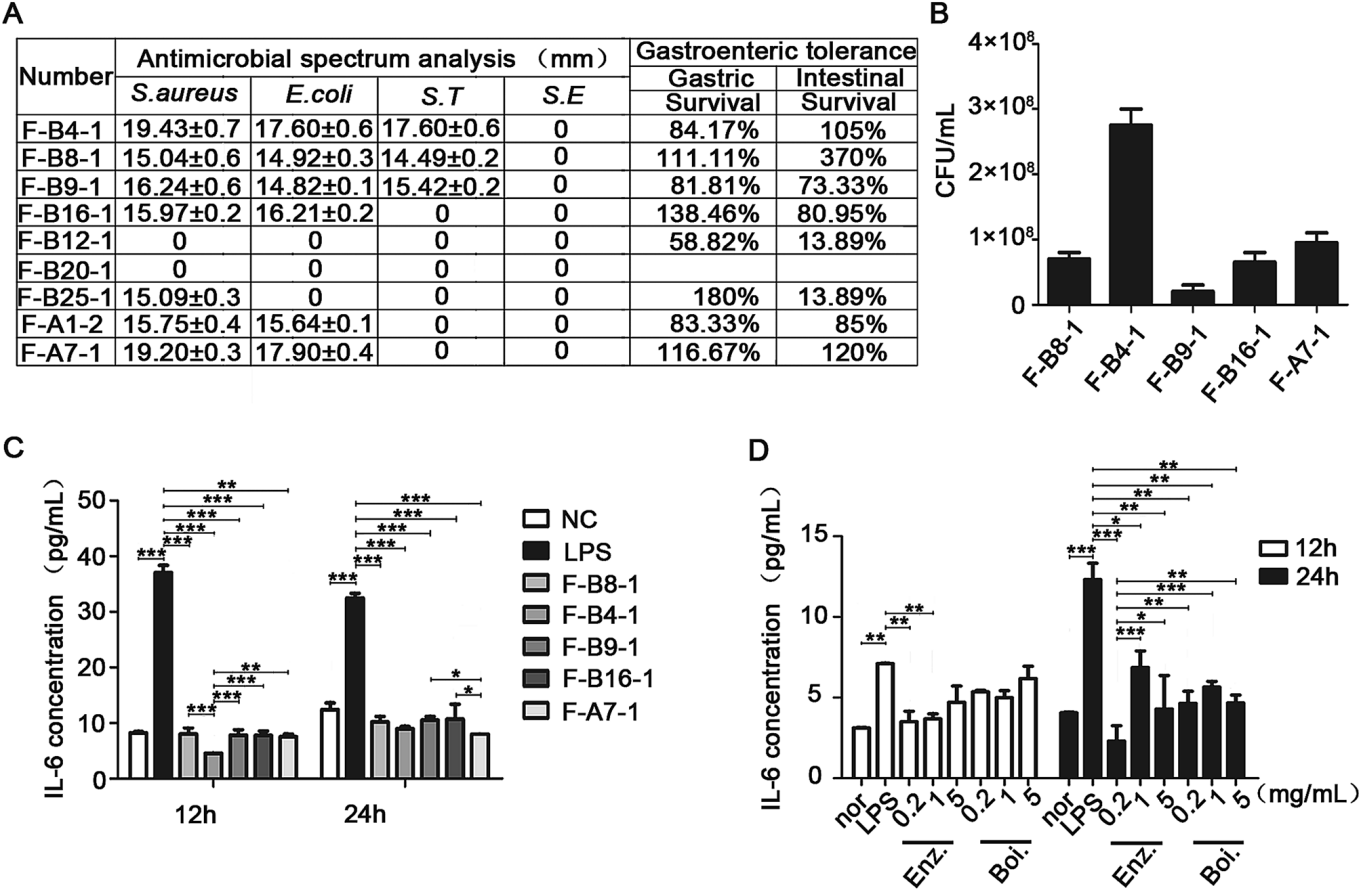
Screening of probiotics and comparison of two traditional extraction methods of Danshen. (**A**) Antibacterial spectrum analysis of probiotics and gastrointestinal fluid tolerance. F-B4-1 (*Lactobacillus Rhamnosus*), F-B8-1 (*Lactobacillus Plantarum*), F-B9-1 (*Lactobacillus Fermenti*), F-B16-1 (*Lactobacillus Casei*), F-B12-1 (*Streptococcus Thermophilus*), F-B20-1 (*Leuconostoc Mesenteroides*), F-B25-1 (*Lactobacillus Harbin*), F-A1-2 (*Bacillus Subtilis*), F-A7-1 (*Bacillus Subtillis Natto*). (**B**) The CFU was detected after cultured for 24 h. (**C**) IL-6 levels were examined in the supernatant of Caco-2 cells which were pretreated with 50 ug/mL LPS for 12 h, then treated with sterilized probiotic liquid for 12 h or 24 h. All groups (NC, F-B8-1, F-B4-1, F-B9-1, F-B16-1, F-A7-1) were statistically analyzed compared with the LPS group. Statistical analysis was conducted between F-B4-1 group and other groups (F-B8-1, F-B9-1, F-B16-1, F-A7-1) at 12 h. Statistical analysis was conducted between F-A7-1 group and other groups (F-B8-1, F-B4-1, F-B9-1, F-B16-1) at 24 h. (**D**) Enz. (enzymolysis), Boi. (boiled). IL-6 levels were examined in the supernatant of Caco-2 cells which were pretreated with 50 μg/mL LPS for 12 h, then treated with different concentrations of the two traditional extraction methods of Danshen for 12 h or 24 h. All groups (Nor, Enz., Boi.) were statistically analyzed with the LPS group. At 24 h, 0.2 mg/mL Enz. group was compared with other treatment groups. (Data are expressed as means ± S.E., *p < 0.05, **p < 0.01 and ***p < 0.001).

Firstly, 9 different probiotics in our laboratory were selected to do the antimicrobial spectrum analysis. The data showed that F-B12-1 and F-B20-1 did not have the antimicrobial ability for all the four pathogens *S. aureus*,* E. coli*,* S. Typhimurium and S. Enteritidis.* All of these probiotics did not have the antimicrobial ability for *S. Enteritidis.* The F-25–1 is bacteriostatic only against *S. aureus*. The F-B16-1, F-A1-2 and F-A7-1 have antimicrobial activity against *S. aureus* and *E. coli*. The F-B4-1, F-B8-1 and F-B9-1 showed antibacterial activity against *S. aureus*, *E. coli* and *S. Typhimurium*. The diameters of F-B4-1 against *S. aureus*, *E. coli* and *S. Typhimurium* were 19.43 ± 0.7 mm, 17.60 ± 0.6 mm and 17.60 ± 0.6 mm, respectively. The diameters of F-A7-1 against *S. aureus* and *E. coli* were 19.20 ± 0.3 mm and 17.90 ± 0.4 mm, respectively. And the diameters of F-B4-1 and F-A7-1 against *S. aureus*, *E. coli* and *S. Typhimurium* were longer than other probiotics. Then the gastric and intestinal fluid tolerance analysis were did. The results showed that the viability of F-B12-1 and F-B25-1 were just 13.89% in the artificial intestinal juice (Fig. 1A). Probiotics need to colonize and survive in the intestine before they reach the intestine and play function. Thus probiotics must be tolerant to the effects of gastric acid, pepsin and trypsin^41,42^. Probiotics colonization and tolerance to artificial gastroenteric fluid have been used to determine the survival rate in the upper gastrointestinal tract, which is also one of the important indicators of probiotics screening^43^. The level of tolerance for choosing probiotics is higher than 70%^44^. Thus, the probiotics of F-B4-1, F-B8-1, F-B9-1, F-B16-1 and F-A7-1 were selected for the further experiment to examine the CFU (Fig. 1B). A large number of studies have shown that after probiotics enter the digestive system, their viable bacteria content must reach more than 1 × 10^6^ CFU/mL to play their health effects. Therefore, the number of viable bacteria in fresh probiotics products should not be less than 1 × 10^6^ CFU /mL to compensate for the loss of viable bacteria when probiotics pass through the human gastrointestinal tract^45,46^. Our data suggested that the probiotics of F-B4-1, F-B8-1, F-B9-1, F-B16-1 and F-A7-1 were all reach this level. The CFU of these five probiotics was all higher than 1 × 10^6^ CFU /mL.

LPS could induce inflammatory injury in caco2 cells which is a recognized cell model for UC^47^. Caco2 cells were treated with different concentrations of LPS for 6 h, 12 h or 24 h. The levels of IL-6 were examined in the cell supernatant. The data showed that the level of IL-6 could be increased significantly after treated with 50 μg/mL LPS for 12 h (Supplemental fig. 1). Thus, we used 50 μg/mL LPS in our next experiments. After caco2 cells were treated with LPS for 12 h, F-B8-1, F-B4-1, F-B9-1, F-B16-1 and F-A7-1 (5 mg/mL) were added to examine the IL-6 concentration. It is suggested that all of these five probiotics could decrease the IL-6 concentration induced by LPS. However, after treated for 12 h, there was a statistical significance among F-B4-1 group and other four probiotic groups. And after treated for 24 h, there was a statistical significance among F-B16-1, F-B9-1 and F-A7-1 probiotic groups. Thus, among these five probiotics, F-B4-1 and F-A7-1 had the best effects and were selected for the further experiments (Fig. 1C).

In order to explore which technology has the better effect of anti-inflammation, Danshen was boiled or enzymatic hydrolyzed to examine the IL-6 concentration in caco2 cells treated with LPS. After treated for 12 h, there was no statistical significance among boiled groups and LPS group (*P* > 0.05). And after treated for 24 h, there was a statistical significance among 0.2 mg/mL of enzymatically hydrolyzed Danshen group and other boiled or enzymatic hydrolyzed groups (Fig. 1D). The results showed that enzymatic hydrolyzed Danshen at 0.2 mg/mL had the best effect to decrease the IL-6 concentration. Based on above results, enzymatic hydrolyzed Danshen and F-B4-1 and F-A7-1 were selected.

### Effects of different fermentation methods on LPS-mediated upregulation of TNF-α, IL-6 and IL-1β

**Figure 2:**
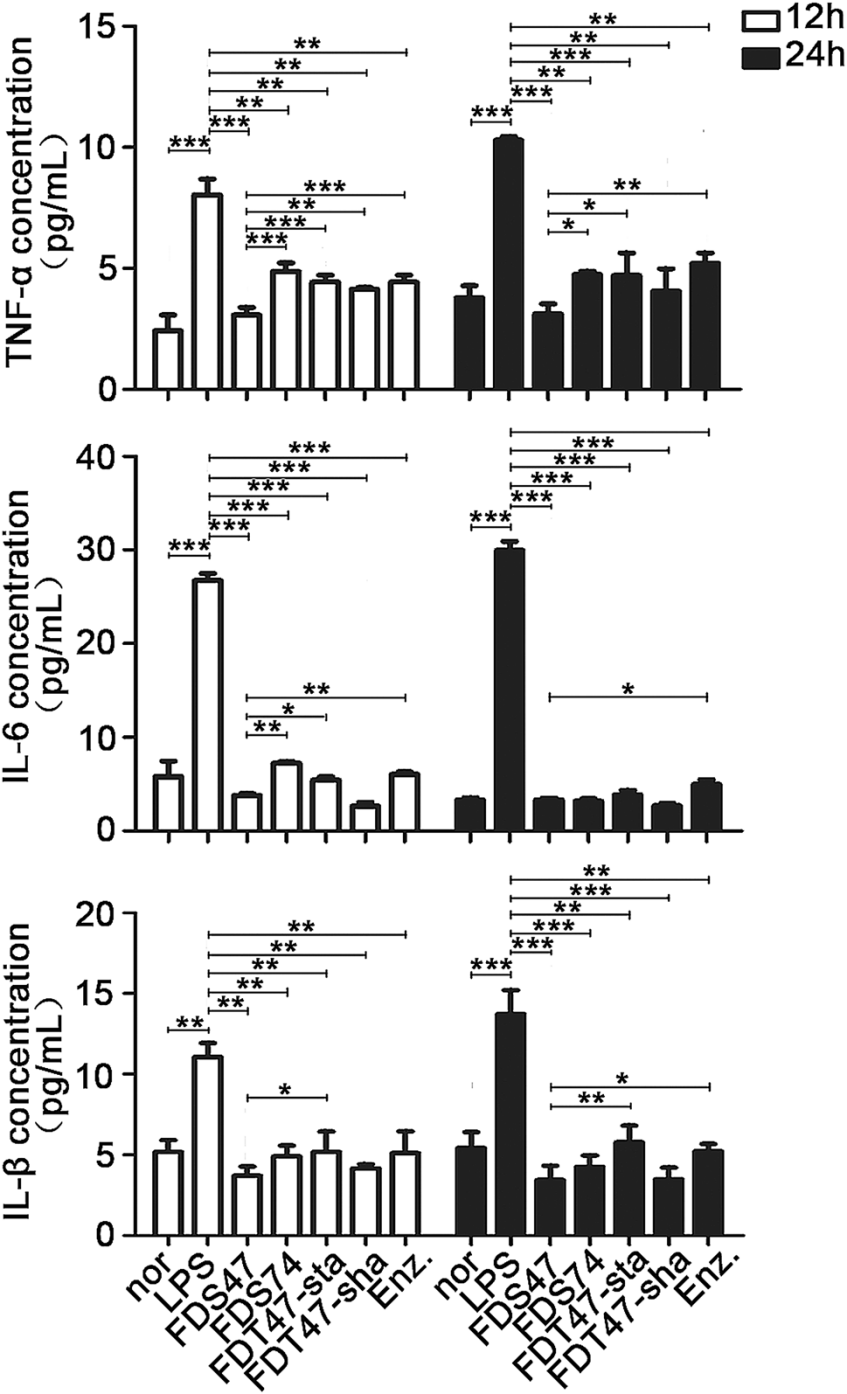
The levels of TNF-α, IL-6 and IL-1β in caco-2 cells after treated with different Danshen products. FDS47 (fermented Danshen sequence 47), FDS74 (fermented Danshen sequence 74), FDT47-sta (fermented Danshen together 47 shake), FDT47-sha (fermented Danshen together 47 stationary), Enz. (enzymolysis). TNF-α, IL-6 and IL-1β levels were examined in the supernatant of Caco-2 cells which were pretreated with 50 ug/mL LPS for 12 h, then treated with different Danshen products for 12 h or 24 h. All groups (Nor, FDS47, FDS74, FDT47-sta, FDT47-sha, Enz.) were statistically analyzed with the LPS group. Statistical analysis was conducted between FDS47 group and other groups (FDS74, FDT47-sta, FDT47-sha, Enz.). (Data are expressed as means ± S.E., *p < 0.05, **p < 0.01 and ***p < 0.001).

**Figure 3:**
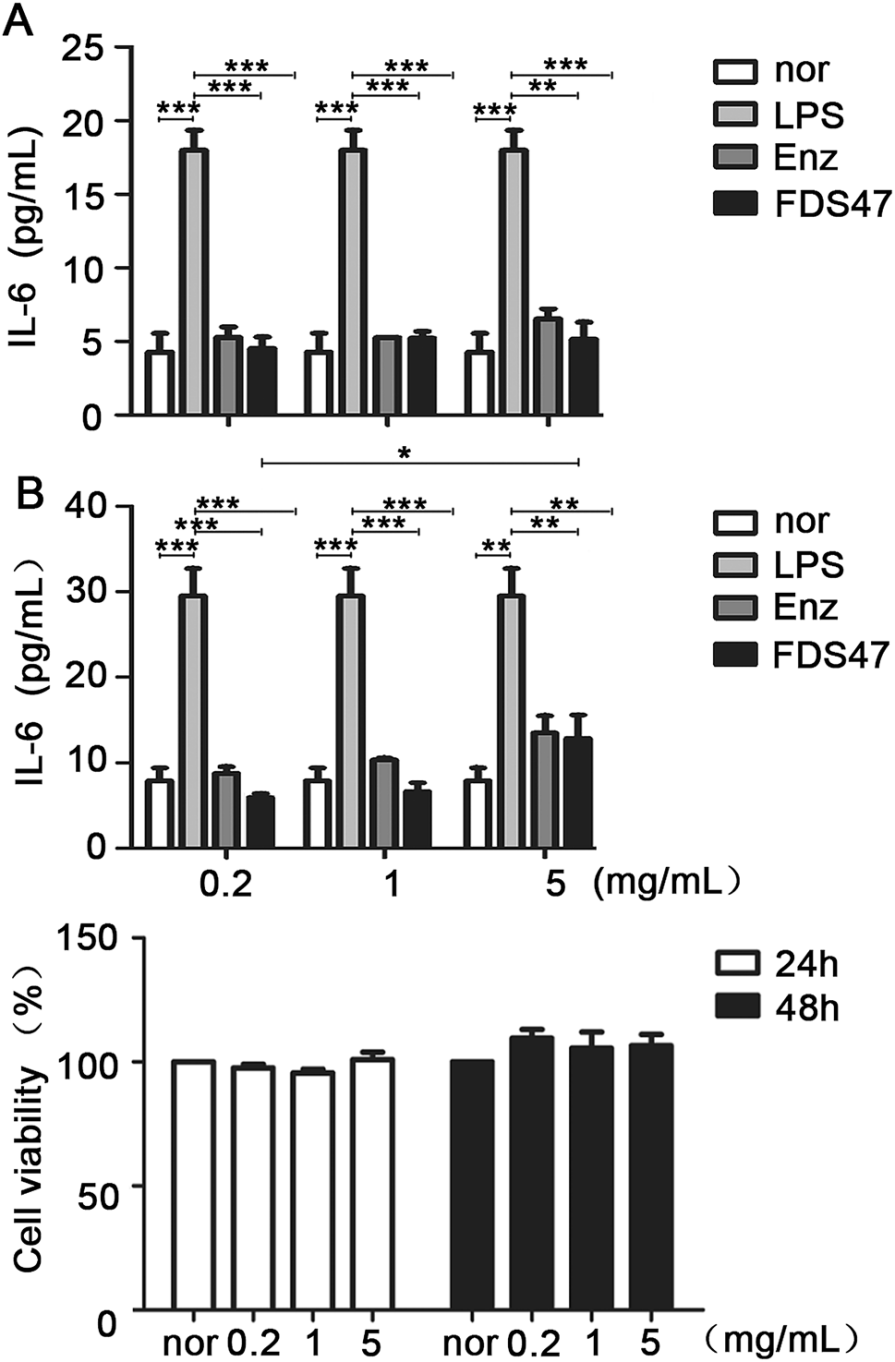
The IL-6 levels in caco-2 cells treated with different concentrations of Enz. and FDS47. FDS47 (fermented Danshen Sequence 47), Enz. (enzymolysis). IL-6 levels were examined in the supernatant of Caco-2 cells which were pretreated with 50 μg/mL LPS for 12 h, then treated with different concentrations of Enz. and FDS47 for 12 h (**A**) or 24 h (**B**). (**C**) The viability of caco-2 cells treated with different concentrations of FDS47. All groups (Nor, Enz., FDS47) were statistically analyzed with the LPS group. Group-to-group statistical analysis was performed at 24 h between 0.2 mg/mL FDS47 group, 1 mg/ mL FDS47 group and 5 mg/mL FDS47 group. (Data are expressed as means ± S.E., *p < 0.05, **p < 0.01 and ***p < 0.001).

ELISA was done to measure the levels of TNF-α, IL-6 and IL-1β in caco2 cells after treatment. The levels of TNF-α, IL-6 and IL-1β were all upregulated after treated with LPS. All of the samples from different fermentation methods could decrease the all three inflammatory factor levels (Fig. 2). Among these different fermentation methods, FDS47 had the best effects of inflammatory factor inhibition. And the effects of inflammatory factor inhibition was better than that in the enzymolysis groups (Fig. 2). There was a statistical significance of TNF-α level among FDS47 group and other treatment groups. As the level of IL-6, there was a statistical significance among FDS47 group and FDS74/FDS-sta/Enz groups. As the level of IL-1β, there was a statistical significance among FDS47 group and FDS-sta/Enz groups (Fig. 2). Thus, together these results, FDS47 had the best effects of inflammatory factor inhibition. Meanwhile, the CFU were examined in F-B4-1 and F-A7-1 with different fermentation methods. There was a statistical significance for the CFU of F-B4-1 among FDS47 group and FDS74/FDT47-sta groups. Although there was no difference between FDS47 and FDT47-sha group in the CFU of F-B4-1 (*P* > 0.05), there was a statistical significance for the CFU of F-A7-1 among FDS47 group and FDT47-sha/FDT47-sta groups. It is suggested that the CFU was higher in FDS47 group (Supplemental fig. 2). Thus, fermented Danshen with F-B4-1 and then with F-A7-1 was selected to do further research. The ELISA results showed that FDS47 at 0.2 mg/mL could inhibit the IL-6 level induced by LPS significantly. With the increased concentrations (5 mg/mL), the IL-6 level elevated in caco2 cells treated with FDS47 for 24 h (Fig. 3A,B). Furthermore, the cell viability was investigated. FDS47 at different concentrations could not cause the changes in cell viability which suggested that FDS47 did not have the cytotoxicity of caco-2 (Fig. 3C).

### FDS47 alleviated the clinical symptoms of DSS-induced colitis in mice

**Figure 4:**
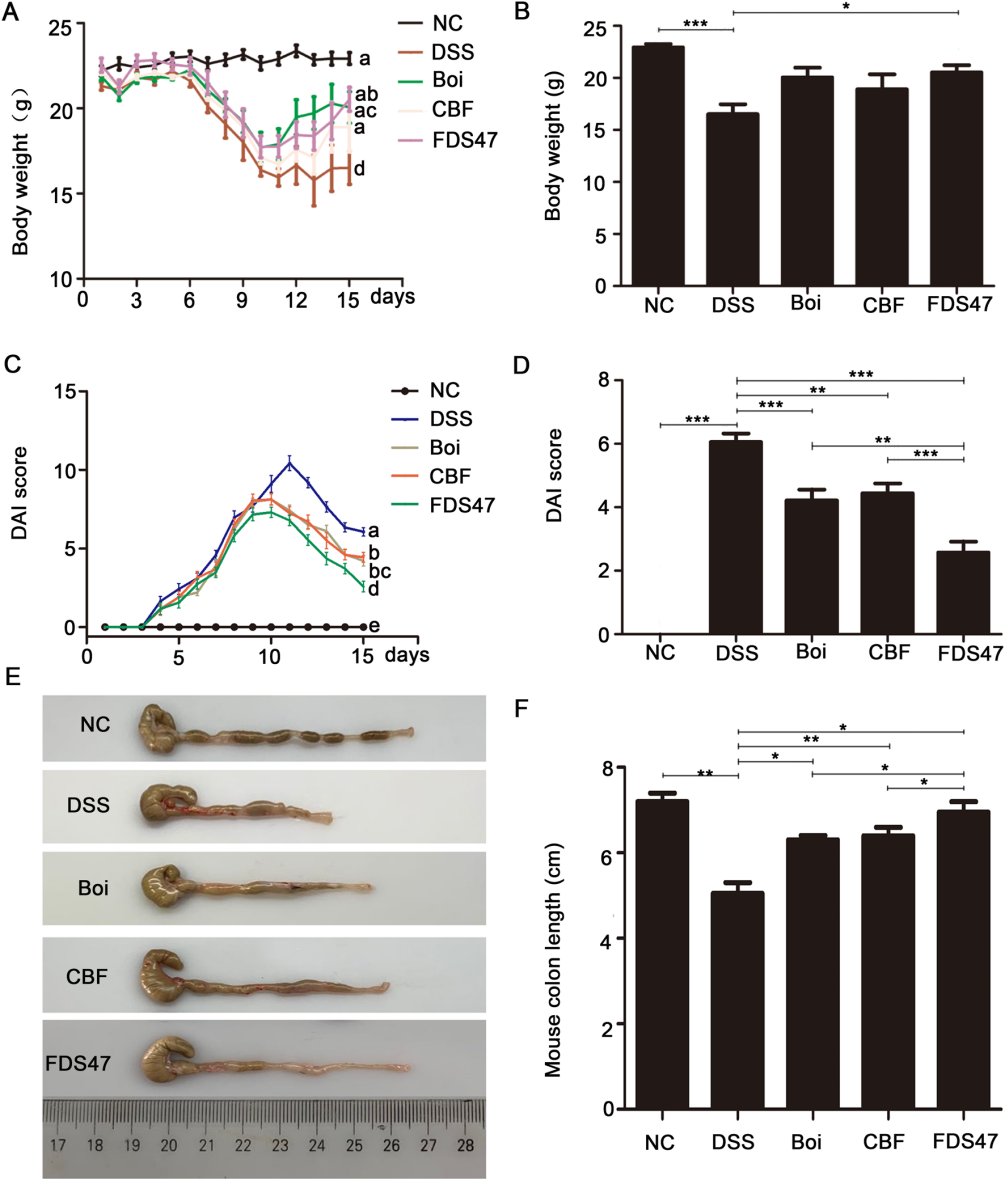
The effects of FDS47 on relieving DSS-induced UC mice was better than boiled Danshen itself or bacteria solution itself. NC (Negative control), FDS47 (fermented danshen sequence 47), Boi. (boiled), CBF (compound bacterium fluid). (**A**) The body weights of mice were detected every day. a-d represented significant differences among different groups by one-way ANOVA procedure followed (*p* < 0.05). (**B**) The statistical analysis of the body weights at the 15th day. (**C**) DAI (disease activity index) score of mice was examined every day. a-e represented significant differences among different groups by one-way ANOVA procedure followed (*p* < 0.05). (**D**) The statistical analysis of the DAI score at the 15th day. (**E**) The photos of colon length (one representative colon from each group). Images were processed by use of Adobe Photoshop SC6 (6.1, https://www.adobe.com/cn/products/photoshop.html) (**F**) The colon lengths calculated from E. All groups (NC, Boi., CBF, FDS47) were statistically analyzed with the DSS group. The FDS47 group and other groups (Boi., CBF) were statistically analyzed from group to group. (Data are expressed as means ± S.E. ^#^*p* > 0.05, *p < 0.05, **p < 0.01 and ***p < 0.001).

We first examined the organs (heart, spleen, kidney, lung and liver) of mice in each treatment group. There was no significant changes in all organs (Supplemental fig. 3). The body weight and colon length are important indicators of colitis severity. DSS-induced colitis could decrease the body weight and shorten the colon length. The data showed that FDS47 could effectively prevented body weight loss induced by DSS (Fig. 4A). At the 15th day, only the body weight in FDS47 group was much higher than that in DSS group (*P* < 0.05) (Fig. 4B). The grade of UC induced by DSS was evaluated by the DAI score, which was the sum of scores given for body weight loss, stool consistency, and presence of fecal blood. A significant increase of DAI score was observed in the DSS-treated group at the 15th day compared with the normal groups (*p* < 0.05). In three treatment groups, the DAI scores were significantly decreased when compared with the DSS group at the 15th day (Fig. 4C). And FDS47 most effectively prevented DAI increase compared with Boi and CBF groups (Fig. 4D). In addition, mean colon length was the highest in the normal mice and lowest in the DSS-induced colitis mice (Fig. 4E, F). In all three treatment groups, the colon lengths were longer than those in the DSS group. In the FDS47 group, the colon length was longer than that in Boi and CBF groups (*P* < 0.05) which almost returned to the normal level (Fig. 4F). As can be seen from body weight, DAI and colon length results above, the effect of FDS47 in the treatment of colitis is better than those of Boi and CBF.

**Figure 5:**
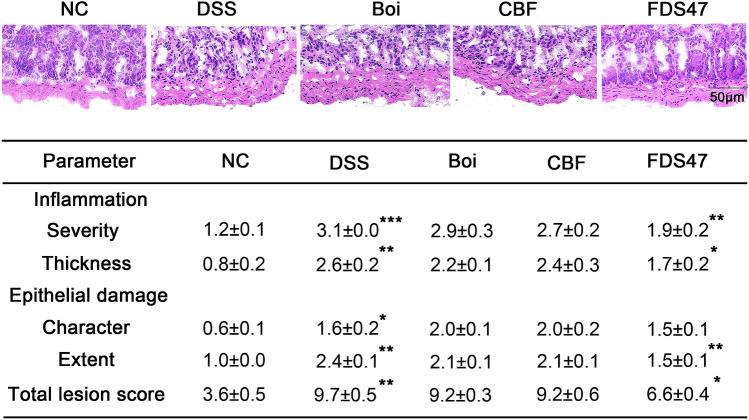
Histological sections of colonic tissue stained with hematoxylin and eosin. (**A**) Histological sections of colonic tissue stained with hematoxylin and eosin (HE) under microscope. Boi (boiled), CBF (compound bacterium fluid) and FDS47 (fermented Danshen sequence 47). (**B**) Effects of DSS on colon pathology of DSS-induced UC mice. DSS group was compared with NC group. FDS47 group was compared with DSS group. (Data are expressed as means ± S.E. *p < 0.05, **p < 0.01 and *** *p* < 0.001).

H&E staining of the colon revealed the degree of inflammation and epithelial damage. The colons from all of the mice in each group were examined in H&E stained slides. According to the Fig. 5A, DSS treated mice displayed the most severe infiltration of inflammatory cells, disruption of surface epithelium, and loss of crypts. Intragastric administration of FDS47 showed the least severe colitis compared with the DSS treated group. H&E scores in all the mice were determined at the 15th day. The total lesion scores in DSS group increased compared with the normal group. And the decrease of the total lesion scores were the most remarkable in the FDS47 group (Fig. 5B). All these above results indicated that FDS47 could significantly alleviate the clinical symptoms of DSS-induced colitis in mice. And the alleviate effects in FDS47 group were better than those in Boi and CBF groups.

### FDS47 decreased pro-inflammatory cytokines in DSS-induced mice

**Figure 6:**
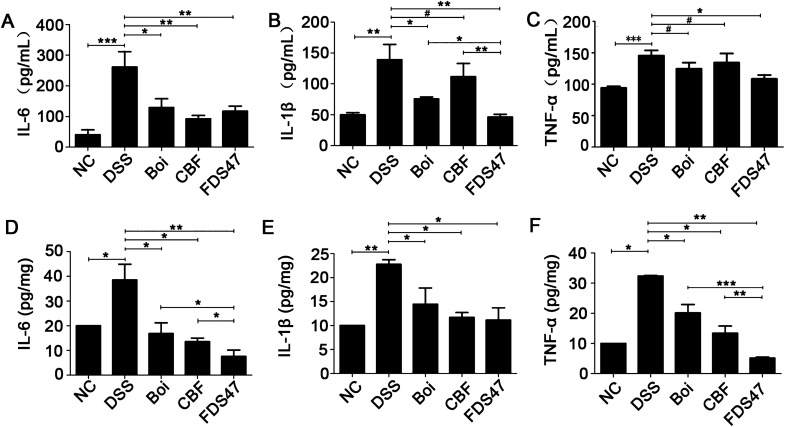
Effects of Boi, CBF or FDS47 on the levels of inflammatory cytokines in mice. Boi (boiled), CBF (compound bacterium fluid) and FDS47 (fermented Danshen sequence 47). The levels of IL-6 (**A**), IL-1β (**B**) and TNF-α (**C**) in serum of DSS- induced UC mice were detected using ELISA Kits. The levels of IL-6 (**D**), IL-1β (E) and TNF-α (F) in the colonic homogenate of DSS-induced UC mice were detected using ELISA Kits. All groups (NC, Boi., CBF, FDS47) were statistically analyzed with the DSS group. The FDS47 group and other groups (Boi., CBF) were statistically analyzed from group to group. (Data are expressed as means ± S.E. ^#^*p* > 0.05, *p < 0.05, **p < 0.01 and ***p < 0.001).

To understand the mechanism that underlined the alleviation of DSS-induced colitis in mice after treatment with FDS47, the levels of pro-inflammatory (TNF-α, IL-6, and IL-1β) in serum (Fig. 6A–C) and colon tissues (Fig. 6D–F) were examined. No matter in serum or colon tissues, the levels of IL-6, IL-1β and TNF-α were significantly increased in DSS treatment group (Fig. 6). After intragastric administration of FDS47, a remarkable decrease of the three pro-inflammatory cytokines were observed. There were statistical differences among FDS47 group and Boi/CBF groups in the levels of IL-1β in serum. And there were statistical differences among FDS47 group and Boi/CBF groups in the levels of IL-6 and TNF-α in the colonic homogenate of DSS-induced UC mice (Fig. 6). Thus, FDS47 was better than Boi and CBF.

## Discussion

Probiotics could relieve various inflammatory diseases by regulating the microbiota in gastrointestinal tract, especially in UC treatment^48^. Nowadays, many probiotics have been shown to have therapeutic effects on UC. Usually, these products are multi-component probiotic mixtures, because each strain may have additive or synergistic effects^49,50^. It is reported that the supplement of *L. plantarum* ZDY2013 and *B. bifidum* WBIN03 could remit UC through modification of gut microbiota to regulate oxidative stress and inflammatory mediators^51^. By the analysis of antibacterial spectrum, gastrointestinal fluid tolerance, bacterial viability and inflammatory factor inhibition, F-B4-1 and F-A7-1 were selected in this work. They could not only effectively reduce the growth of several harmful bacteria, but also have good tolerance to the gastrointestinal environment (Fig. 1A,B). Additionally, the metabolites of these two probiotics could significantly reduce the release of inflammatory factors in caco-2 cells caused by LPS (Fig. 1C). From these results, it is suggested that F-B4-1and F-A7-1could become hopeful strains in curing UC.

In China, Herbs have been used for thousands of years to cure of diseases. Until now, a large number of biologically active substances in herbs have been proved^52^. Danshen is a very valuable herb in Chinese traditional medicine. It has been reported that Danshen has the antioxidant, anti-inflammatory and antibacterial activities^53^. The anti-inflammation efficacy of Danshen is generally played by the main biological activities in it, such as Salvianolic acid B, tanshinone IIA, Danshensu and dihydrotanshinone I^54^. Thus, Danshen was selected as fermentation material for the treatment of UC in this work. The traditional extraction methods for Danshen were boiling water extraction and enzymatic hydrolysis. Compared with boiling water extraction, enzymatic hydrolysis could better reduce the release of cellular inflammatory factors (Fig. 1D). Thus, we selected enzymatic hydrolysis to deal with Danshen to release more biological activities.

It is reported that the therapeutic effects of fermented herbs were better than that of herb itself. KIOM-MA is a specific agent for allergic and chronic inflammatory diseases, which is composed of several plants, included *Glycyrrhizae radix*,* Polygoni cuspidati radix*,* Sophorae radix*,* Cnidii rhizoma*, and *Arctii fructus*. Recently, it is proved that the KIOM-MA128, the probiotics fermentation product of KIOM-MA had the improved therapeutic efficacy via the absorption and bioavailability of the active ingredients^55^. FRAM, the fermented products of Rhizoma Atractylodis Macrocephalae (RAM), also exerted a better protective effects on intestinal epithelial cells (IECs) against LPS-induced perturbation of membrane resistance and permeability^56^. The metabolic processes could be improved in herbs fermented with probiotics^57^. And in the process of fermentation, the decomposition of organic matter could be promoted by microorganisms and many new micromolecules could be produced from macromolecules^58^. It is reported that fermentation could better extract the effective ingredients in Chinese herbal medicines to treat enteritis^52^. Therefore, we used the selected probiotics F-B4-1and F-A7-1to ferment Danshen for the first time. The data showed that the fermented Danshen had the better effects than boiled Danshen itself or bacteria solution itself on UC treatment, including the colon length recover, decreased DAI score, lung damage recover and anti-inflammatory activities (IL-6, IL-1β and TNF-α) in serum and colons in DSS induced UC mice (Figs. 4, 5, 6). It is suggested that after fermentation, Danshen and probiotics might interact synergistically. However, the synergetic mechanisms, whether effective ingredients was increased or the new biological components were produced after fermentation, need to be further investigated in the next step.

At present, although it is believed that probiotics are safe under normal circumstances, there are individual reports of local or systemic infections such as pericarditis and sepsis caused by the ingestion of certain *lactobacilli*,* bifidobacteria* and other *lactic acid* bacteria^59^. Especially for patients with immunodeficiency, broken bowel syndrome, central duct occlusion, heart valve disease or premature infants, the risk of adverse events might be higher if probiotics are taken indiscriminately^60^. In severely UC, due to the destruction of the integrity of the intestinal barrier, there is a danger of live probiotics from the intestines and stomach to the internal organs of the body (bacterial displacement), which may lead to bacteremia^61^. Due to the dangers of probiotics during use, we choose safer metabolic fermentation products and verify its safety in in vitro cell experiments and in vivo experiments in mice.

It has been pointed out that the reduction of inflammatory cytokines in the serum and colons represents a logical target for UC therapy^62^, such as TNF-α, IL-6 and IL-1β, which play leading roles in the formation of UC^3,63^. Our data suggested that after intragastric administration of the fermentation products, all these pro-inflammatory cytokine (TNF-α, IL-6 and IL-1β) levels were decreased in the serum and colons of the DSS-induced UC mice (Fig. 6). Also, the in vitro experiments showed it. The fermentation products inhibited the pro-inflammatory cytokine (TNF-α, IL-6 and IL-1β) levels in the caco-2 cells treated by LPS (Fig. 2). All of the results proved that the probiotics fermented Danshen products relieved the DSS-induced UC mice by blocking pro-inflammatory cytokines.

## Conclusions

**Figure 7:**
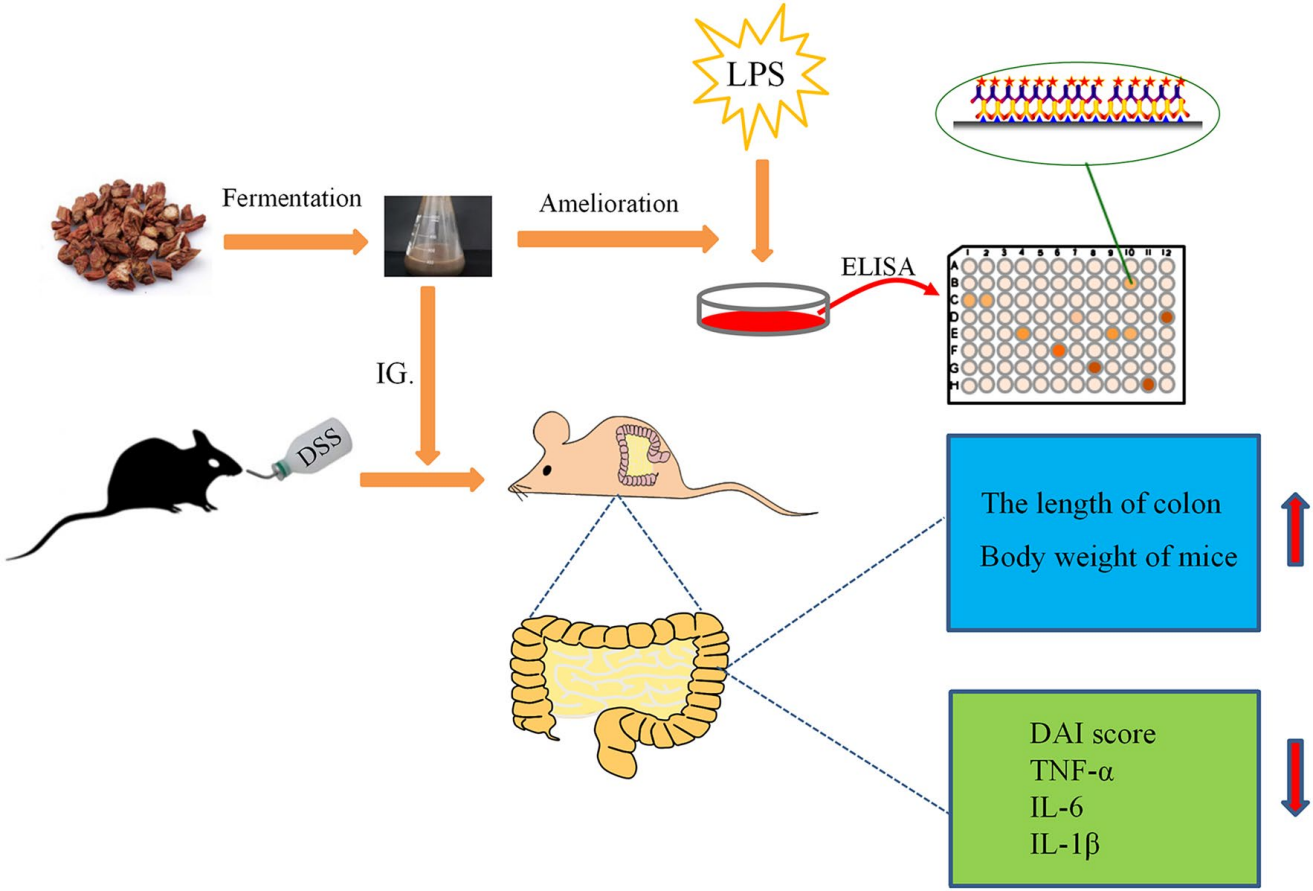
The diagram of the experimental procedure and results. Images were processed by use of Adobe Photoshop SC6 (6.1, https://www.adobe.com/cn/products/photoshop.html) and Scienceslides 2016 (1.16.06.16, https://www.visiscience.com/scienceslides) of Microsoft office professional plus 2013 (15.0.4420.1017, https://www.microsoft.com/zh-cn/download/details.aspx?id=54146).

In summary, we screened F-B4-1and F-A7-1 from many probiotics which could reduce harmful bacteria growth, have tolerance to the gastrointestinal environment and inhibit the inflammatory factors caused by LPS to ferment enzymatic Danshen. The fermentation products has anti-colitis effects through the inhibition of pro-inflammatory factors (Fig. 7). And compared with Danshen, fermented Danshen relieved DSS-induced UC in mice more effectively. Thus, the fermented Danshen might be further developed as an effective treatment approach to treat intestinal inflammation.

## Supplementary Information

Supplementary Information.

## Data Availability

The datasets generated during and/or analyzed during the current study are available from the corresponding author on reasonable request.
