# Supplementary material for: Fermentation products of Danshen relieved dextran sulfate sodium-induced experimental ulcerative colitis in mice
Source: Sci Rep. 2021 Aug 10;11:16210. doi: 10.1038/s41598-021-94594-7 (PMC8355158; doi:10.1038/s41598-021-94594-7)
Supplement: Supplementary file 1 — Supplementary Information. [file 41598_2021_94594_MOESM1_ESM.docx]

**
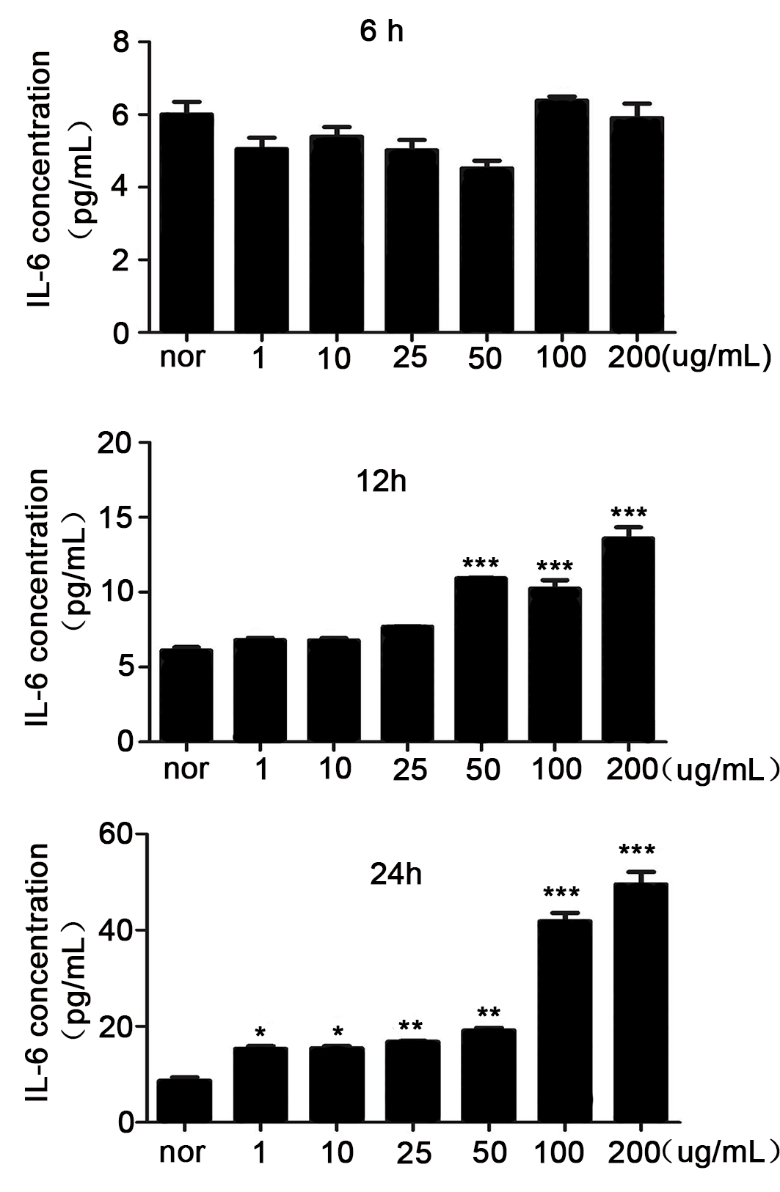
**

**Supplemental figure 1. Establishment of the LPS induced caco-2 cell model of UC in vitro.** The supernatant of caco-2 cells treated with different concentrations of LPS were collected at 6 h, 12 h or 24 h. The IL-6 levels were detected using ELISA kits. (Data are expressed as means ± S.E. **p* < 0.05, ***p* < 0.01 and ****p* < 0.001 *v.s.* normal group)


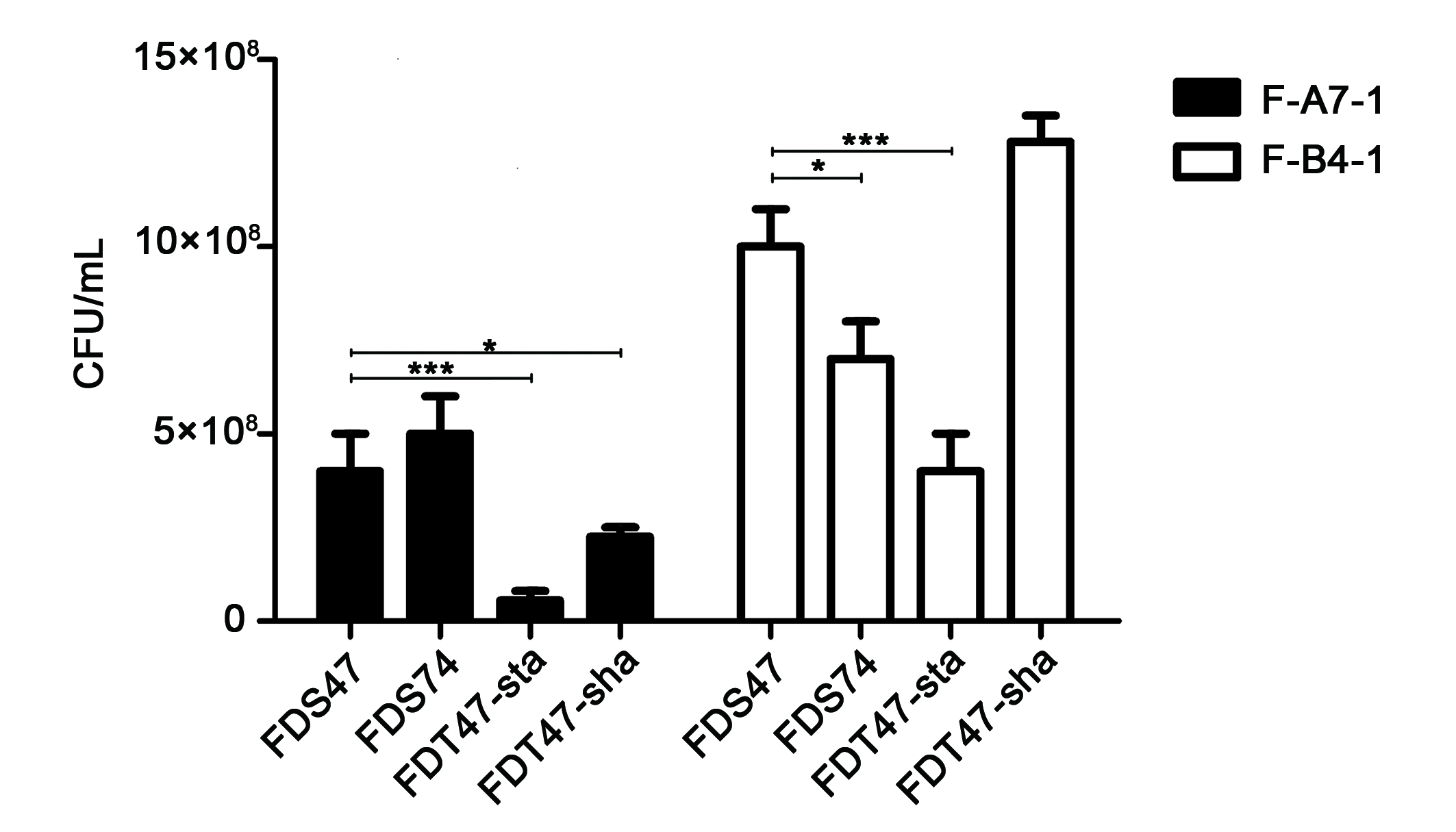


**Supplemental figure 2. Maximum viable count.** FDS47 (fermented Danshen sequence 47), FDS74 (fermented Danshen sequence 74), FDT47-sta (fermented Danshen together 47 shake), FDT47-sha (fermented Danshen together 47 stationary). The supernatant of the fermented Danshen liquid was obtained. The plates were incubated at 37 ℃ for 24 h. And the viable cells were enumerated by the plate count method. Cell counts were expressed as CFU/mL.


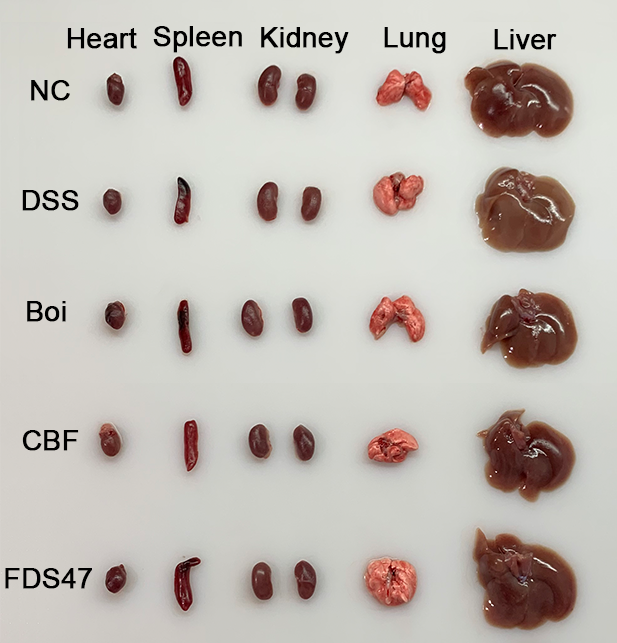


**Supplemental figure 3. Photographs of hearts, spleens, kidneys, lungs and livers in DSS-induced UC mice treated with Boi (boiled), CBF (compound bacterium fluid) or FDS47 (fermented Danshen sequence 47).**
